# Hard and Soft Tissue Relapse After Different Genioplasty Procedures: A Scoping Review

**DOI:** 10.7759/cureus.41478

**Published:** 2023-07-06

**Authors:** Munish Kumar, Rachel S Singh, Gagandeep Singh, Pritam Raj, Himanshi Gupta, Rishabh Kasrija

**Affiliations:** 1 Department of Oral and Maxillofacial Surgery, Guru Nanak Dev Dental College and Research Institute, Sunam, IND; 2 Department of Oral and Maxillofacial Surgery, JSS Dental College and Hospital, Mysuru, IND

**Keywords:** genioplasty, scoping review, chin, relapse, reduction, advancement

## Abstract

The chin is a crucial component of facial aesthetics, and 20% of craniofacial problems require repair of the chin size, shape, and position. Genioplasty is used to treat irregularities in all three planes of the chin. Specific hard and soft tissue relapses following various genioplasty techniques have not been adequately studied in the literature to date. The purpose of this scoping review was to investigate the stability of hard and soft tissue changes achieved by different genioplasty procedures, six months after the procedure. A literature search was performed on PubMed, Web of Science, Embase, Wiley Online, Scopus, Google Scholar, Science Direct, and Cochrane databases from January 1, 2011 to October 31, 2022. Prospective and retrospective cohorts, case-control studies, observational studies, and randomized control trials, with at least 10 patients, which were written in English and evaluated the stability of different genioplasty procedures, with a follow-up period of at least six months were included. The manual and electronic search yielded 523 articles, and after complete screening, seven articles were selected (five with advancement genioplasty and two with reduction genioplasty) that met the eligibility criteria for review. The patients undergoing reduction genioplasty had a mean age of 24.15 years, compared to 20.5 years for augmentation genioplasty. The average follow-up period was 18.64 months for augmentation genioplasty and 10.5 months for reduction genioplasty technique. The relapse was assessed at pogonion, and it was noted that the average surgical advancement at hard tissue pogonion was 7.04 mm with a relapse of 0.69 mm after six months post-treatment. The average vertical movement of the hard tissue pogonion was 1.8 mm with a relapse of 0.74 mm. The average reduction at hard tissue pogonion was 3.2 mm in the vertical direction with a relapse of 0.2 mm and 0.8 mm reduction in soft tissue pogonion with a relapse of 0.3 mm. The soft to hard tissue ratio mentioned in the different studies ranged from 0.89 to 0.97. Both reduction and augmentation genioplasty are stable and reliable for altering the chin position for aesthetic purposes. The recommended mode of fixation is rigid fixation.

## Introduction and background

Aesthetics plays a very important role in human life, regardless of the era. The shape, size, and position of the chin are pivotal in providing facial harmony. Genioplasty is a surgical procedure aimed at altering the position and/or shape of the chin to improve facial aesthetics and function. The genial area can be modified in all three planes of space to alter the chin and improve the well-being of the individual [1]. Genioplasty addresses a variety of chin-related concerns, including recessive chin, protrusive chin, and asymmetry. Several different techniques have been tried over the years, including horizontal sliding genioplasty, genioplasty with implants, and various other modifications of the conventional types, in order to achieve anterior-posterior reduction, vertical reduction, asymmetry correction, and widening or narrowing of the posterior and anterior dimensions of the chin [2]. The specific technique used depends on the individual needs and goals of the patient.

Chin advancement genioplasty was first performed by Otto Hofer in 1942 on a cadaver [3]. Later, various surgical techniques involving either an intraoral or extraoral approach to repositioning the chin in all three planes of space were introduced using rigid fixation or wire fixation [4]. Recently, the use of computer-assisted and virtual surgical planning techniques in genioplasty has increased. These methods allow for a precise and individualized surgical plan and can help minimize surgical complications and improve outcomes [2]. Like any surgical procedure, genioplasty also carries the risk of relapse and stability issues during long-term follow-up [5]. Therefore, it is crucial to assess the relapse rate of different genioplasty procedures in both hard and soft tissue areas. Only one systematic review has been conducted to assess the long-term stability of advancement genioplasty [6].

Owing to the lack of sufficient data, the primary objective of this review article was to evaluate the long-term stability and relapse associated with different genioplasty procedures. The secondary objective was to relate it to the amount of advancement or reduction and the method of fixation. A preliminary search of the Cochrane Database and JBI Evidence Synthesis was conducted, and it was found that no systematic or scoping reviews on this topic were performed.

## Review

Methods

The PRISMA Extension for Scoping Reviews (PRISMA-ScR) guidelines and the JBI protocol were followed in this review to provide clear and transparent reporting of the outcomes [7]. Ethical committee approval was not needed, as this review was conducted on an online database. Scoping reviews, unlike systematic reviews, are not registered at the International prospective register of systematic reviews (PROSPERO). The study selection for this review was established by using the PICO approach:

P (Population/patients) - Humans of all ages who have undergone genioplasty procedures with at least six months follow-up.

I (Intervention) - Advancement or reduction genioplasty procedure for correction of the chin.

C (Comparator) - Comparisons were made between the preoperative, immediately postoperative, and long-term postoperative skeletal and soft tissue structures.

O (Outcome) - The primary outcome measure was to evaluate the hard and soft tissue relapse at the level of pogonion after six months of treatment and, secondarily, to assess the relationship between relapse and the amount of surgical movement.

Literature search

**Figure 1:**
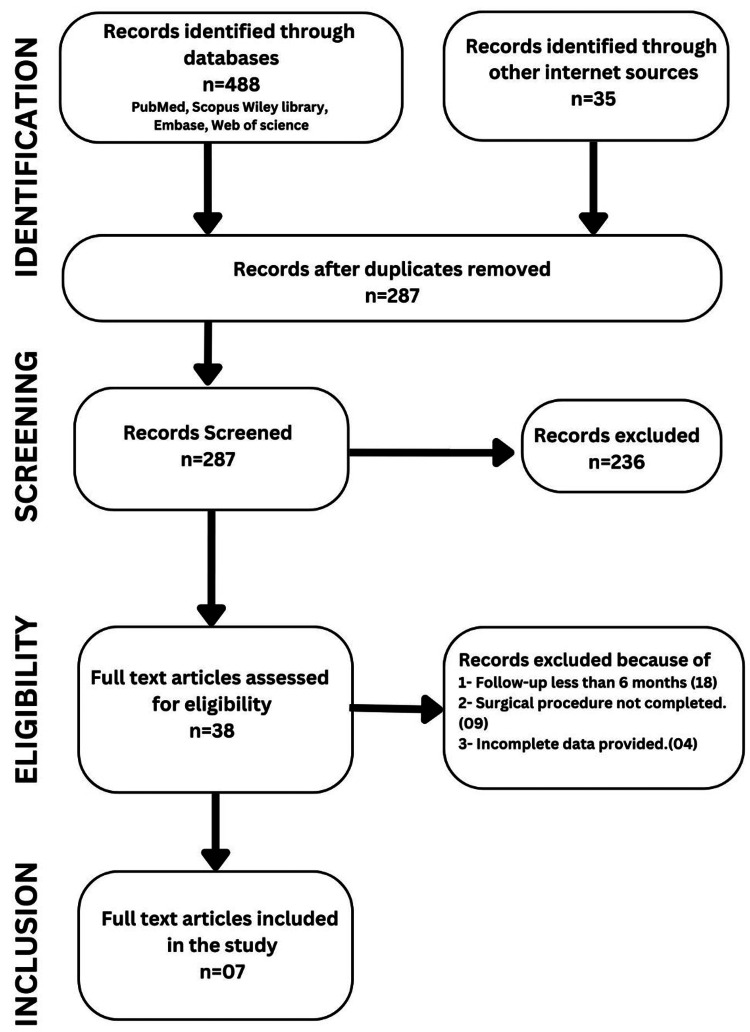
PRISMA-ScR flow diagram for the data search PRISMA-ScR - PRISMA Extension for Scoping Reviews

A systematic search of the English literature was conducted in PubMed, Free E-Journals, Wiley Online Library Journals, Google Scholar, Web of Science, Scopus, Embase, and Cochrane electronic database for articles published between January 1, 2011 and October 31, 2022, using Medical Subject Heading Term: “stability or recurrence or relapse” (All Fields) AND “genioplasty or chin surgery or chin advancement or chin reduction” (All Fields). The PRISMA-ScR flow diagram of the data search is shown in Figure 1.

Data collection

Inclusion Criteria

All retrospective and prospective cohort studies, case-control studies, observational studies, and randomized control trials, involving humans of all ages and any sex, with a follow-up of at least six months, wherein genioplasty was performed as an isolated procedure, between January 2011 and October 2022, and written in English were included.

Exclusion Criteria

Case series, theses, letters to the editor, editorials, case reports, animal studies, systematic reviews, meta-analyses, follow-up periods of less than six months, genioplasty with any other procedure, studies published before January 2011 and after October 2022, and studies published in any language other than English were excluded.

Data extraction

After performing manual and electronic searches in all the mentioned databases and grey literature, the studies were independently assessed for eligibility based on titles and abstracts by two reviewers (MK, RS). After removing duplicates, the full-text articles were assessed in detail against the inclusion criteria, and the reasons for further exclusion of articles were recorded and reported in this scoping review. Any disagreements between reviewers at each stage of the selection process were resolved through discussion until a consensus was reached. Information on patient demographic characteristics, type of genioplasty procedure, amount of surgical movement, hard and soft tissue changes during surgery, and relapse at pogonion during a follow-up period of more than six months.

Results

Initial electronic and manual searches using keywords yielded a total of 523 articles. After removing duplicates, 287 articles were selected for review by two independent reviewers. Thirty-eight articles were found to be eligible for full-text assessment, out of which 31 articles were further rejected due to the use of additional surgical procedures apart from genioplasty or an insufficient follow-up period of less than six months, and seven articles were included in this review (Figure 1) [1,5,8-12].

Detailed Analysis of the Studies

The descriptive analysis of the studies showed that the total number of studies in augmentation genioplasty was five with 139 patients, and chin reduction studies included two with 589 patients. The average age of patients in augmentation genioplasty was 20.5 years, and chin reduction was 24.15 years. All studies had more females than males (2:1). The type of fixation used in these studies was rigid. The average follow-up period was maximum in augmentation genioplasty (18.64 months) and less in the reduction genioplasty technique (10.5 months), as shown in Table 1.

**Table 1:** Demographic data of the studies M: Male, F: Female, Retro: Retrospective cohort, Prosp: Prospective cohort, Adv.: Advancement, Red.: Reduction

S No.	Authors	Year	Type of study	No. of patients	Age (years)	M	F	Treatment	Type of surgery	Type of fixation	Follow-up (Months)
1	Erbe et al. [5]	2011	Retro	14	26.1	3	11	Adv. Genioplasty	Sliding	Rigid	12
2	Reddy et al. [8]	2011	Retro	10	22	4	6	Adv. Genioplasty	Horizontal	Rigid	9.2
3	Kim et al. [9]	2014	Retro	97	26.7	15	82	Red. Genioplasty	Vertical	Rigid	9
4	Kumar et al. [10]	2015	Retro	15	19.1	6	9	Adv. Genioplasty	Sliding	Rigid	24
5	Budharapu et al. [1]	2017	Prosp	25	19.1	6	19	Adv. Genioplasty	Sliding	Rigid	24
6	Park et al. [11]	2019	Retro	40	21.6	23	17	Red. Genioplasty	Sliding	Rigid	12
7	Chamberland et al. [12]	2022	Prosp	75	16.1	25	50	Adv. Genioplasty	Sliding	Rigid	24

Advancement Genioplasty

Five studies reported up-to-date data regarding hard and soft tissue relapse at the pogonion in the horizontal and vertical directions at six months or more of the post-treatment follow-up period [1,5,8,10,12]. The average horizontal surgical advancement at hard tissue pogonion was 7.04 mm with a relapse of 0.69 mm after six months post-treatment. The average vertical movement of hard tissue pogonion was 1.8 mm with a relapse of 0.74 mm after at least six months. Only one study reported a significant relapse in the vertical direction [5] and one study in the horizontal direction [10]. The mean surgical advancement at soft tissue pogonion in the horizontal direction was 6.9 mm with a relapse of 1.27 mm; in the vertical direction it was 1.8 mm change with a 0.8-mm relapse during follow-up. The soft to hard tissue ratio mentioned in the different studies ranged from 0.89 to 0.97. No correlation was observed between the amount of advancement and relapse. The mode of fixation was rigid in all the studies (Table 2).

**Table 2:** Changes in hard and soft tissue variables following genioplasty (mm) -ive sign: reduction or setback, Pg: Pogonion, adv.: Advancement, NA: Not assessed

S No.	Authors	Hard tissue changes at pogonion (mm)					Soft tissue changes at pogonion (mm)				Soft tissue to hard tissue ratio
		Vertical		Horizontal		Vertical			Horizontal		
		Pg adv.	Pg relapse	Pg adv.	Pg relapse	Pg adv.		Pg relapse	Pg adv.	Pg relapse	
1	Erbe et al. [5]	3	1.6	7.9	0.1	2.1		1.6	10.5	2.9	0.97
2	Reddy et al. [8]	0.28	0.21	7.92	0.85	1.19		0.89	6.35	1.84	0.89
3	Kim et al. [9]	-3.5	-0.23	NA	NA	-1.9		-1.4	NA	NA	NA
4	Kumar et al. [10]	2.1	1.1	6.2	1.5	0.6		0	4.8	1	0.77
5	Budharapu et al. [1]	.3.76	0.28	6.8	0.08	.3.44		0.4	6.4	0.28	0.9
6	Park et al. [11]	-3.38	-0.22	-3.16	-0.5	-0.48		-0.19	-0.98	-0.2	0.94
7	Chamberland et al. [12]	1.7	0.54	6.39	0.91	1.7		0.3	6.72	0.33	0.92

Reduction Genioplasty

Only two studies were found in which isolated reduction genioplasty was performed without any additional surgical procedures [9,11]. Both studies reported no significant changes during the follow-up period after the procedure. Only one study reported changes in hard and soft tissue pogonions in both the horizontal and vertical directions [11]. The average reduction at hard tissue pogonion was 3.2 mm in vertical direction with a relapse of 0.2 mm and 0.8 mm reduction in soft tissue pogonion with a relapse of 0.3 mm. The horizontal reduction of 3.16 mm with a relapse of 0.5 mm in hard tissue pogonion and 0.98 mm horizontal reduction in soft tissue pogonion with a relapse of 0.2 mm was noted in one study. No correlation was observed between reduction and relapse rates. The fixation mode used was rigid (Table 2).

Discussion

Scoping reviews are a relatively new approach that provides insights into the current evidence available on a particular topic. Their methodology is almost the same as that of a systematic review, but it does not produce critically appraised and synthesized results; therefore, it does not require assessment of methodological limitations and risk of bias, unlike a systematic review [13].

It is critical to achieve an ideal chin shape during genioplasty. There is little information in the literature about the long-term effects of genioplasty as a standalone procedure. No systematic or scoping reviews have evaluated the stability of alterations in hard and soft tissues following isolated advancement and reduction genioplasty accessible in the literature. Two different mechanisms could have an impact on osteotomy of the inferior border of the mandible: the first is the shift in the position of the moved genial segments before complete osseous union, leading to skeletal instability; and second is the osseous remodeling, where the advanced genial segment undergoes a slow remodeling process, impacting the stability of the changes achieved at the time of surgery [14]. Therefore, a longer follow-up period is needed to evaluate the stability of changes achieved with genioplasty. In our study, the average follow-up period was 18.6 months for advancement genioplasty and 10 months for reduction genioplasty.

Genioplasty is usually performed as an additional procedure along with bilateral sagittal split osteotomy (BSSO) of the mandible; therefore, it is difficult to assess the changes produced by genioplasty alone. Only seven studies were available in the literature that performed isolated genioplasty procedures and had a follow-up period of at least six months. Most of these were retrospective studies that used rigid fixation [5,8-11]. Rigid fixation offers stability in large advancements and shows good adaptability in three dimensions. After the movement of the genial segments, it is very important to hold them rigidly at that position to prevent relapse and resorption. Therefore, it is preferred over wire fixation [14,15].

After analyzing every study, it was found that there was no association between the rate of improvement or reduction and the rate of relapse throughout the follow-up period. The initial relapse might have been caused by biological reactions to the surgery, and it was not tied to how much improvement or reduction had been made. Most studies have revealed the stability of changes with no significant relapse of hard and soft tissues. Only two studies have reported soft tissue relapse at the pogonion in the horizontal direction [10] and hard tissue relapse in the vertical direction [5]. Only advancement genioplasty was affected by relapse, whereas reduction genioplasty was unaffected. The intermittent pulling of the hyoid muscles, which causes them to stretch out during the progress of genial segments, may be the likely cause of this, which then causes relapse and osseous site resorption. To evaluate the stability of the results after therapy, it is crucial to understand the mechanism by which soft tissues react with hard tissues. The ratio ranged from 0.89 to 0.97:1, which was good performance. The mentalis muscle plays a significant role in proper soft tissue chin contour post-operatively, as it is the muscle which provides vertical support to the lower lip, and if not positioned properly, can lead to the poor soft tissue to hard tissue response, and formation of “witch’s chin” [15]. The reason for the variation found in the studies might be differences in patient demographic characteristics, such as age. Age is an important factor in determining bone remodeling at the surgical site [16]. Reduction genioplasty leads to an increased amount of soft tissue chin and better response, compared to advancement genioplasty, where tension is created at the soft tissue chin, leading to less response. Therefore, it is very important to consider individual soft tissue variations during surgery planning to minimize relapse and maximize facial aesthetics.

Most studies have used pogonion point as a reference to study changes in the procedure. According to Defreitas et al. [17], the pogonion is a stable landmark of the chin and can still be considered an important factor in genioplasty studies despite the controversy surrounding it [18]. The post-surgical stability of pogonion can also be due to the rigid fixation, which has been used in all the studies [19].

Our review revealed that most of the patients who opted for isolated genioplasty procedures were females (2:1) and in the younger age group (20.5-25.6 years). The reason for this is the growing awareness among people about improving facial aesthetics, where genioplasty is performed as a cosmetic procedure. Most patients have dental Class I with skeletal Class II or III jaw relationship with a recessive or very prominent chin, respectively, affecting the facial profile [6]. Few participants in the research reported surgical problems such as intraoral dehiscence of the incision, infection, hemorrhage, devitalization of the teeth, lip paresthesia, and chin ptosis, but each was effectively treated with minimal intervention [6]. Basic knowledge about the teeth, osseous and neurovascular anatomy in the area, and careful planning will circumvent significant complications. To avoid complications during the procedure, certain precautions have to be taken such as maintaining the vascular supply of hard tissue chin by leaving the caudal portion attached to the soft tissue chin, and by having correct knowledge of origin and insertion of geniohyoid, genioglossus, mylohyoid, mentalis and mentolabial muscles [20].

Limitations

Owing to the heterogeneity of the studies, this scoping review serves as a foundation for further in-depth systematic reviews and meta-analyses that are necessary in this situation. Additionally, the majority of trials in which genioplasty was performed as an independent procedure were very limited and retrospective in nature, and the sample size was small. In place of 2D lateral cephalograms, 3D imaging should be employed to evaluate the outcomes. Future prospective studies with a large sample size and long-term follow-up are needed, as are 3D imaging methods for the evaluation of outcomes.

Clinical implications

Based on our scoping review, both advancement and reduction genioplasty are recommended procedures to improve facial aesthetics, with good post-treatment stability and minimal complications.

## Conclusions

Without requiring the relocation of tooth-bearing structures, horizontal bony osteotomy of the chin offers the advantage of greatly improving facial skeletal aesthetics. This is a flexible method for addressing both excessive and insufficient chin abnormalities. According to the existing studies, the following conclusions can be drawn on the effectiveness of genioplasty treatment in the long term: There was a good soft tissue-to-hard tissue response and good stability with little relapse, there was no connection between the amount of advancement or reduction and recurrence, and a rigid type of fixation was advised. Both advancement and reduction genioplasty procedures yield predictable and stable results. The need for well-designed randomized controlled trials and prospective studies with proper reporting of patient-related demographic data and long-term follow-up is quite evident from this review.
